# Concurrent Use of Hypnotic Drugs and Chinese Herbal Medicine Therapies among Taiwanese Adults with Insomnia Symptoms: A Population-Based Study

**DOI:** 10.1155/2013/987862

**Published:** 2013-09-24

**Authors:** Kuei-Hua Lee, Yueh-Ting Tsai, Jung-Nien Lai, Shun-Ku Lin

**Affiliations:** ^1^Institute of Traditional Medicine, School of Medicine, National Yang-Ming University, Linong Road, Taipei 112, Taiwan; ^2^Department of Pharmacy, Taipei City Hospital, Songde Branch, Taipei 110, Taiwan; ^3^Department of Chinese Medicine, Taipei City Hospital, Yangming Branch, Taipei 111, Taiwan; ^4^Department of Chinese Medicine, Taipei City Hospital, Renai Branch, Taipei 106, Taiwan

## Abstract

*Background*. The increased practice of traditional Chinese medicine (TCM) worldwide has raised concerns regarding herb-drug interactions. The purpose of our study is to analyze the concurrent use of Chinese herbal products (CHPs) among Taiwanese insomnia patients taking hypnotic drugs. *Methods*. The usage, frequency of services, and CHP prescribed among 53,949 insomnia sufferers were evaluated from a random sample of 1 million beneficiaries in the National Health Insurance Research Database. A logistic regression method was used to identify the factors that were associated with the coprescription of a CHP and a hypnotic drug. Cox proportional hazards regressions were performed to calculate the hazard ratios (HRs) of hip fracture between the two groups. *Results*. More than 1 of every 3 hypnotic users also used a CHP concurrently. *Jia-Wei-Xiao-Yao-San* (Augmented Rambling Powder) and *Suan-Zao-Ren-Tang* (Zizyphus Combination) were the 2 most commonly used CHPs that were coadministered with hypnotic drugs. The HR of hip fracture for hypnotic-drug users who used a CHP concurrently was 0.57-fold (95% CI = 0.47–0.69) that of hypnotic-drug users who did not use a CHP. *Conclusion*. Exploring potential CHP-drug interactions and integrating both healthcare approaches might be beneficial for the overall health and quality of life of insomnia sufferers.

## 1. Introduction

Inadequate sleep and sleep disorders are common, and are associated with an increased risk of poor health, diminished work and academic performance, and negative safety outcomes that have critical clinical and economic ramifications [1]. Although substantial progress has recently been made in the pharmacologic treatment of insomnia, including the use of benzodiazepines and Z-drugs (zaleplon, zolpidem, and zopiclone), adverse effects and the potential for abuse, addiction, and the development of tolerance have led to poor compliance for hypnotic regimens among insomniac patients [2, 3]. Many poor sleepers have turned to traditional Chinese medicine (TCM) remedies to manage their symptoms because they believe that such treatments exert fewer subjective residual effects [4].

Although TCM remedies are promoted as natural, and therefore harmless, complementary, and alternative medicines that can also be used in Western countries [5, 6], data are limited regarding their safety when used in combination with hypnotic drugs, particularly regarding the risk of herb-drug interactions, such as that resulting from interference with the clearance of either of the drugs. Studies on the prevalence of hypnotic-drug use and the prescription patterns of TCM remedies among hypnotic-drug users are scant. 

Comprising unique traditional therapies for various ailments, TCM has been used in Taiwan for hundreds of years, and its popularity remains unabated, despite the present availability of modern medical care in Taiwan. In addition, one distinguishing feature of the national healthcare system in Taiwan is the coexistence of modern Western medicine (WM) and TCM, including acupuncture and manipulative therapies and Chinese herbal products (CHPs), claims for which have been covered by the National Health Insurance (NHI) system since 1995 [7]. 

People in Taiwan are free to choose from care offered by WM clinics or TCM clinics. With an insured rate of 98% to 99%, the random sample that comprises the NHI research database (NHIRD) is representative of the general population of Taiwan and should allow a reasonably accurate assessment of the concurrent usage of TCM and modern medical resources in Taiwan; therefore, the NHIRD provides an ideal platform for pharmacoepidemiological studies [8]. Our study aimed to describe the demographics and patterns of CHP usage among hypnotic-drug users and, by using a large population-based retrospective database, explore the risk of hip fracture among a cohort of hypnotic-drug users prescribed with a CHP concurrently compared with hypnotic-drug users who did not use Chinese herbs. Our findings provide evidence-based information for formulating appropriate management strategies for drug safety and integrative medicine. 

## 2. Materials and Methods

### 2.1. Data Source and Participants

Our study protocols were approved by the Institutional Review Board of the Committee on Chinese Medicine and Pharmacy (CCMP), Department of Health, Taiwan. Our population-based study retrospectively analyzed the reimbursement records of 1 million NHI beneficiaries in the NHIRD that had been previously selected at random from the 22 million beneficiaries of the NHI to determine the prevalence of concurrent CHP and hypnotic use between January 1, 2002, and December 31, 2008, in Taiwan. The electronic records of the NHIRD use beneficiary identification numbers that are encrypted and maintained by the National Health Research Institutes (NHRI) of Taiwan [9, 10]. 

The NHIRD records contain demographic information, including age and sex, and clinical data, including all records of clinical visits and hospitalizations, and all information regarding prescribed drugs and dosages, including those for CHPs. The diagnoses used in the NHIRD are coded according to the International Classification of Diseases, Ninth Revision, Clinical Modification (ICD-9-CM) [11]. 

Because the cost of all prescribed hypnotic drugs is reimbursed by the NHI, they cannot be dispensed at a pharmacy without a physician's prescription. To construct a fixed cohort, all outpatients with insomnia who used hypnotic drugs, including benzodiazepines, zolpidem, and zopiclone, were reviewed. For the purpose of studying the use of CHPs, we downloaded the claims forms for reimbursed CHPs from the website of the Bureau of National Health Insurance. The corresponding information regarding the CHP was then obtained from the CCMP website, including the name of each herb, the proportion of each constituent of the mixture, the date and period of approval for the drug, the CCMP manufacturer code, and the name of the CHP manufacturer. All CHPs with the same CCMP standard formula are classified in the same category, regardless of slight variations in the products among different CHP manufacturers [12]. For our analysis of the demographic and clinical variables, a coprescription of a CHP and a hypnotic drug (defined as prescriptions for both issued simultaneously or issued separately with overlapping treatment periods) was used to determine the use of a CHP and a hypnotic drug on the same day.

**Figure 1:**
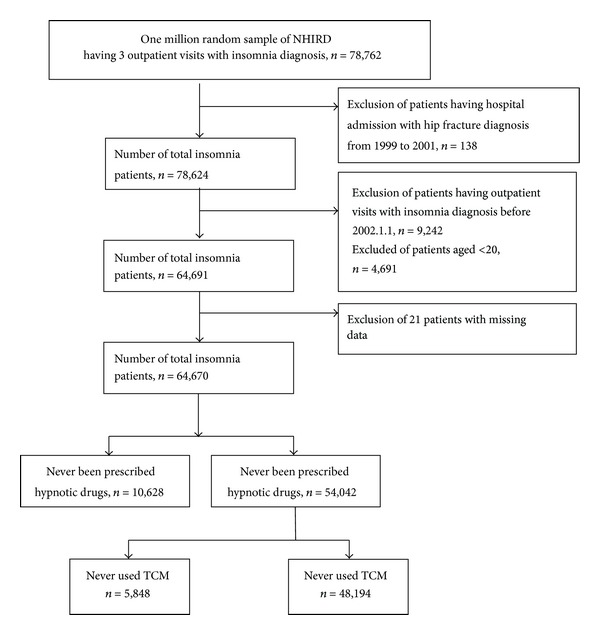
Flowchart of recruitment of subjects with insomnia from the 1 million random sample of the National Health Insurance Research Database (NHIRD) from 2002 to 2008 in Taiwan.

The participant selection from the NHIRD was performed as shown in Figure 1. We reviewed all beneficiaries with at least three outpatient visits with any nonorganic insomnia (ICD-9-CM codes 307.40-42, 307.45-49, 780.50-52, and 780.55-59) and excluded patients who were admitted to hospital and received a hip fracture diagnosis between 1999 and 2001 (*n* = 138). Patients with hyperinsomnia (ICD-9-CM codes 327, 307.43, 307.44, 780.53, and 780.54) for the calendar year in which it occurred were excluded. The prevalent insomnia cases diagnosed before the end of 2001 (*n* = 9,242) and patients under 20 years of age (*n* = 4,691) were excluded to ensure that all participants had been newly diagnosed with adult insomnia. Patients with incomplete data for age or sex (*n* = 21) and insomniac patients not taking hypnotic drugs (*n* = 10,628) were also excluded. 

### 2.2. Study Variables

To identify the key factors associated with the coadministration of hypnotics and CHP among insomnia sufferers (CAHCHP as an acronym for this population), we selected the demographic factors according to previous studies [1, 7, 8]. Patients were classified, based on age, into one of seven groups as follows: 20–29 years, 30–39 years, 40–49 years, 50–59 years, 60–69 years, 70–79 years, and ≥80 years. The geographic areas of Taiwan in which patients resided were classified as one of the following seven regions: Taipei city, Kaohsiung city, Northern region, Central region, Southern region, Eastern region, and Outlying islands. Patients' monthly income in New Taiwan Dollars (NT$) was categorized as one of the following four levels: $0, $1–$19,999, $20,000–$39,999, and ≥$40,000.

The variables for hypnotic-drug use included in our analyses were defined according to the specific proprietary hypnotic preparation used during the study period. We categorized the types of preparation used as follows: long-half-life benzodiazepine; short-half-life benzodiazepine; zolpidem; zopiclone; and mixed regimens, including two, three, or more of the aforementioned preparations. 

To estimate the impact of CHP use on the rate of hip fracture, we selected subjects who were first recorded with a diagnosis of hip fracture (ICD-9-CM codes 820.0–820.9) between January 1, 2002, and December 31, 2008. Furthermore, we also analyzed the risk of hip fracture according to the period of time patients were administered hypnotic drugs (≥30 days and <30 days).

### 2.3. Statistical Analysis

Data analysis was conducted using descriptive statistics, including the prescription rates of patients' concurrent use of a CHP and a sedative hypnotic drug stratified by age and sex, indications for the prescribed CHP, and the most frequently coprescribed herbal formulas for treating insomnia. The indications were coded according to the ICD-9-CM and grouped into different broader disease categories. The ICD-9-CM codes 460–519 were classified as diseases of the respiratory system. Codes 780–799 were grouped as symptoms, signs, and ill-defined conditions, and codes 520–579 were classified as diseases of the digestive system. Multiple logistic regression was conducted to evaluate factors that correlated with CHP use based on the odds ratio (OR) and the 95% confidence interval (CI). Cox proportional hazards regressions were performed to calculate the hazard ratio (HR) of hip fracture among hypnotic-drug users who used a CHP concurrently and hypnotic-drug users who did not use Chinese herbs. A significance level of *α* = 0.05 was selected. The SAS statistical software, version 9.3 (SAS Institute, Cary, NC, USA), was used for data management and analyses. 

## 3. Results

The database of outpatient claims contained information for 64,670 sleep-disturbance sufferers during the 2002–2008 period. A total of 54,042 (83.6%) insomnia patients treated with sedative-hypnotic drugs were included in our study. Most of these patients used short-acting benzodiazepines. Most of the sedative-hypnotic users (89.2%) also sought care from TCM practitioners. Up to 37.0% (*n* = 19,994) of the sedative-hypnotic users received a coprescription for a CHP, resulting in the coadministration of a sedative-hypnotic drug with a CHP. Such patients who were coadministered the two types of drug are hereafter referred to as CAHCHP patients. A total of 19,994 CAHCHP patients were identified in our insomnia cohort.

The hypnotic users who did not use a CHP were significantly older and more likely to be male than were the CAHCHP patients. More CAHCHP patients had income levels of $20,000 to $39,999 and above, resided in Central Taiwan, and used two or more types of sedative-hypnotic drugs than the sedative-hypnotic users who did not use a CHP. The adjusted ORs (aORs) and 95% CIs calculated using the logistic regression model are displayed in Table 1. After adjusting for other factors, the aORs of CAHCHP patients (aORs = 2.49) were higher for women than for men (aORs = 1.00) except patients aged 80 years and older. Compared with the patients aged 40–49 years (aORs = 1.00), the aORs of the CAHCHP patients decreased with age. Patients who used at least two types of sedative hypnotic drugs (two types: OR = 1.30; 95% CI: 1.20–1.42 and ≥ three types: OR = 1.90; 95% CI: 1.75–2.06) were more likely to be CHP users than were the patients who used only one sedative hypnotic drug. 

By analyzing the percentage distribution of the 19,994 CAHCHP patients, we observed that sleep disturbance (1,450,671 TCM visits) was the most common major disease category for coprescription events, followed by diseases of the digestive system (1,349,092 TCM visits) and the diseases of the respiratory system (1,319,859 TCM visits), as summarized in Table 2. As shown in Table 3, zolpidem, alprazolam, lorazepam, estazolam, and fludiazepam were the five sedative hypnotic drugs that were most frequently coprescribed with a CHP. The details of the most frequently used CHP formulas are shown in Table 3. *Suan-Zao-Ren-Tang* (Zizyphus Combination) was the most frequently used CHP, followed by *Jia-Wei-Xiao-Yao-San *(Augmented Rambling Powder) and *Tian-Wang-Bu-Xin-Dan *(Ginseng and Zizyphus Combination). The average number of days of concurrent use of a sedative hypnotic drug and a CHP was approximately 30 days per CAHCHP patient during the 7-year study period. 

The HRs of hip fracture for CAHCHP patients and hypnotic-drug users who did not use Chinese herbs are presented in Table 4. After adjusting for potential confounders, the HR of hip fracture for CAHCHP patient was 0.57-fold (95% CI = 0.47–0.69) that of comparison subjects. We further analyzed the risk of hip fracture according to the length of hypnotic treatment. Table 4 shows that, compared with hypnotic-drug users who did not use Chinese herbs, the HR of hip fracture for CAHCHP patients who were prescribed hypnotics for ≥30 days was 0.62 (95% CI = 0.50–0.77) and as low as 0.33 (95% CI = 0.20–0.54) for CAHCHP patient who were prescribed hypnotics for <30 days. The incidence rate of hip fracture among the CAHCHP patients was no higher than that of hypnotic-drug users who did not use Chinese herbs (≥30-day group: 3.4 versus 6.5 per 100 person-years; <30-day group: 2.9 versus 11.1 per 100 person-years).

## 4. Discussion

According to our review of the literature, this study is the first to use a random population-based cohort to document the coprescription of hypnotic drugs and CHPs in insomnia patients in Taiwan. We observed that hypnotic use is common among insomnia patients in Taiwan and that benzodiazepines were the most frequently prescribed category of sedative hypnotic, as shown in Table 1. Previous studies have reported that patients treated with benzodiazepines may be at higher risk for falls and have been associated with an increased risk of hip fracture. Both physicians and patients should be aware of the association between hypnotic use and the potential risk for injury in Taiwan. The possibility of recall or selection bias can be excluded because we included patients who were newly diagnosed with insomnia by qualified conventional physicians during the 2002–2008 period from a random sample of the population-based NHI database.

The prevalence of the concurrent use of sedative hypnotics and CHPs was 37% (Figure 1). The abuse of benzodiazepines has become a serious problem in North America [13]. The fear of sedative hypnotic side effects or dependency may motivate patients to seek TCM therapies [2, 3, 14]. Although barbiturates have been reported to be the most prescribed hypnotic in Taiwan, our findings indicate a change in the prescribing habits of physicians, with nonbarbiturates, particularly zolpidem, being coprescribed more frequently than barbiturates. We also observed that poor sleepers who were prescribed multiple types of hypnotics were more likely to use a CHP concurrently compared with patients who did not seek TCM treatment.

In Taiwan, the prescribing of hypnotic drugs must be accompanied by a standard ICD-9-CM diagnosis code [9, 10] to adhere to the requirements for NHI claims reimbursement. The change in prescription patterns that we observed might represent an attempt by physicians to achieve a greater therapeutic effect for patients with a history of tolerance, poor response, or dependence in previous hypnotic-drug treatment [15] or it might reflect an increasing concern among physicians regarding the potential abuse of sedative hypnotic drugs. Considering the increasing incidence of hypnotic-CHP coprescriptions, we suggest that a more critical attitude toward the use of hypnotics and CHPs in combination is required among both physicians and hypnotic-drug users. We observed that hypnotic-drug users who use a CHP concurrently were not more strongly associated with an increased risk of hip fracture than were hypnotic users who did not use Chinese herbs. Little is known regarding the potential interactions of CHPs with hypnotic drugs; therefore, healthcare providers and public-health policy analysts should focus greater attention on the potential long-term impact of this particular healthcare-seeking behavior on health outcomes [16].

Our previous clinical trials demonstrated that *Jia-Wei-Xiao-Yao-San *(Augmented Rambling Powder) and* Suan-Zao-Ren-Tang* (Zizyphus Combination), the two CHPs that were most commonly coprescribed with a hypnotic drug, may be an efficacious therapy for improving sleep quality [5, 6], as shown in Table 3. Among the five most frequently coprescribed formulas for treating insomnia, *Tian-Wang-Bu-Xin-Dan *(Ginseng and Zizyphus Combination), *Gan-Mai-Da-Zao-Tang* (Licorice and Jujube Combination), and *Chai-Hu-Jia-Long-Gu-Mu-Li-Tang *(Bupleurumand Mu Li Combination) all have a long history of use in Taiwan. They are said to nourish the blood and calm the nerves and are frequently prescribed by TCM practitioners to alleviate sleep disturbances [17].

The concomitant use of benzodiazepines and Z-drugs was the most common combination of hypnotics that were used by patients who used multiple types of hypnotics. Because of a lack of sufficient evidence supporting this less judicious prescription pattern, we suggest that such patients may be more likely to “doctor shop,” resulting in the high prevalence of the concurrent use of CHPs and hypnotic drugs. Our findings indicate that the lower incidence rate and HR of hip fracture in CAHCHP patients than that in hypnotic-drug users who did not use Chinese herbs might imply that TCM physicians in Taiwan encouraged insomnia patients to coadminister CHPs and hypnotic drugs to improve sleep quality, which might allow lower hypnotic dosages and reduce the risk of hypnotic adverse effects and dependence. However, insufficient evidence exists to support such a conclusion.

Although previous studies have demonstrated that acupuncture might be an alternative therapy for insomnia [18, 19], our data indicated that insomnia patients typically sought acupuncture treatment for injury, poisoning, and diseases of the musculoskeletal system and connective tissue. Following insomnia, diseases of the digestive system were the second most frequent disease category for TCM visits by CAHCHP patients. These results indicate that health care providers should also address the general health condition of insomnia patients, particularly gastroenterological symptoms, to identify possible causes of insomnia and provide appropriate treatment for other such medical needs that do not require hypnotic treatment. Further studies are warranted to investigate the cost effectiveness of the coadministration of hypnotic drugs with CHPs and comprehensive gastroenterological care for insomnia patients, particularly those with a history of hypnotic dependence.

Our study has three limitations: first, the NHI only reimburses the cost of the CHP; the cost of decoction is not reimbursed, which may have affected patients' decisions to use CHPs. Thus, the frequency of the concurrent use of hypnotic drugs and CHPs may be underestimated in our results. However, because the NHI provides comprehensive coverage and the copayment for prescriptions is always $50 (approximately equal to US $1.50), which is less than the typical cost of herbs sold in Taiwan's markets, the likelihood that patients purchased herbs outside of the NHI system is low. Second, we were unable to draw any conclusions regarding the relationship between the severity of the insomnia and TCM usage because such clinical data are not included in the NHIRD. Third, the retrospective design of our study and the lack of a randomized placebo group might diminish the statistical power of our findings. Thus, our results must be interpreted cautiously because we cannot exclude the possibility of placebo effects.

## 5. Conclusion

Our findings may have implications for the treatment of insomnia patients. Our results suggest that, with equal availability of conventional medical and TCM care, more than one-third of the hypnotic-drug users used CHPs concurrently for the relief of sleep disturbance and gastroenterological symptoms. Recognizing the benefits of TCM, exploring potential interactions and adverse effects, and integrating both healthcare approaches might be beneficial to the overall health and quality of life of insomnia patients, particularly those with a history of hypnotic dependence. Thus, health care providers should proactively explore personalized, optimal detoxification for hypnotic dependence while attending to patients' psychosocial and physical needs. 

## Figures and Tables

**Table 1:** Demographic characteristics and results of multiple logistic regression showing the adjusted odds ratio (aOR) and 95% confidence interval (CI) of patients with newly diagnosed insomnia ever been prescribed hypnotic drugs from the 1 million random sample of the National Health Insurance Research Database (NHIRD) from 2002 to 2008 in Taiwan.

Characteristics	Total no.	Hypnotic users without using Chinese medicine, no. (%)	Hypnotic users coprescribed Chinese medicine, no. (%)	Hypnotic users coprescribed Chinese medicine/hypnotic users without using Chinese medicine	
				Odds ratio	(95% CI)
Numbers of hypnotic users among insomnia population	54,042	5,848 (10.8)	19,994 (37.0)		
Sex					
Male	19,354	3,234 (55.3)	6,350 (31.8)	1.00	(2.34–2.66)
Female	34,688	2,614 (44.7)	13,644 (68.2)	2.49	
Mean (SD) age at inclusion, years	48.8 (15.4)	55.1 (17.2)	49.9 (14.9)		
*Age groups at inclusion, years no. (%) *					
20~29	5,616	362 (6.2)	1,631 (8.2)	1.06	(0.93–1.22)
Male		184 (3.0)	477 (5.8)		
Female		178 (3.2)	1,154 (2.4)		
30~39	11,105	891 (15.2)	3,820 (19.1)	1.03	(0.94–1.14)
Male		506 (6.6)	1,184 (13.2)		
Female		385 (8.6)	2,636 (5.9)		
40~49	13,531	1,193 (20.4)	5,074 (25.4)	1.00	
Male		675 (8.9)	1,536 (17.7)		
Female		518 (11.5)	3,538 (7.7)		
50~59	10,637	1,072 (18.3)	4,184 (20.9)	0.85	(0.77–0.93)
Male		550 (8.9)	1,118 (15.3)		
Female		522 (9.4)	3,066 (5.6)		
60~69	6,657	865 (14.8)	2,798 (14.0)	0.76	(0.68–0.84)
Male		488 (6.5)	962 (9.2)		
Female		377 (8.3)	1,836 (4.8)		
70~79	4,787	922 (15.8)	1,951 (9.8)	0.52	(0.46–0.58)
Male		545 (6.5)	821 (5.7)		
Female		377 (9.3)	1,130 (4.1)		
Over 80	1,709	543 (9.3)	536 (2.7)	0.24	(0.21–0.28)
Male		286 (4.4)	252 (1.4)		
Female		257 (4.9)	284 (1.3)		
*$NT/month (Premiums) *					
0	11,542	1,279 (21.9)	4,735 (23.7)	1.00	
1–19,999	27,391	3,268 (55.9)	10,123 (50.6)	0.85	(0.79–0.92)
20,000–39,999	9,601	729 (12.5)	3,303 (16.5)	1.13	(1.01–1.26)
≥40,000	5,508	572 (9.8)	1,833 (9.2)	1.03	(0.91–1.17)
*Insured area *					
Taipei city	9,495	1,159 (19.8)	3,239 (16.2)	1.00	
Kaohsiung city	3,735	364 (6.2)	1,392 (7.0)	1.46	(1.27–1.68)
Northern Taiwan	14,900	1,848 (31.6)	4,929 (24.7)	0.98	(0.90–1.08)
Central Taiwan	12,554	824 (14.1)	5,490 (27.5)	2.49	(2.24–2.77)
Southern Taiwan	11,910	1,401 (24.0)	4,407 (22.0)	1.28	(1.16–1.41)
Eastern Taiwan	1,078	155 (2.7)	439 (2.2)	1.01	(0.82–1.24)
Outlying islands	370	97 (1.7)	98 (0.5)	0.36	(0.27–0.49)
*Types and prescription patterns of hypnotics *					
Usual treatment regimens	16,182	1,352 (23.1)	3,310 (16.6)	1.00	
Zolpidem	1,623	350 (6.0)	265 (1.3)		
Zopiclone	109	16 (0.3)	20 (0.1)		
BZD-long	8,657	293 (5.0)	1,703 (8.5)		
BZD-short	5,793	693 (11.9)	1,322 (6.6)		
Mixed regimens					
Co-prescribed two types of hypnotics	18,466	2,168 (37.1)	6,734 (33.6)	1.30	(1.20–1.42)
Zolpidem + Zopiclone	79	20 (0.3)	18 (0.1)		
Zolpidem + BZD-long	1,796	275 (4.7)	486 (2.4)		
Zolpidem + BZD-short	4,418	849 (14.5)	1,499 (7.5)		
Zopiclone + BZD-long	136	25 (0.4)	40 (0.2)		
Zopiclone + BZD-short	308	63 (1.1)	99 (0.5)		
BZD-long + BZD-short	11,729	936 (16.0)	4,592 (23.0)		
Coprescribed more than two types of hypnotics	19,394	2,328 (39.8)	9,950 (49.8)	1.90	(1.75–2.06)

NT$ refers to new Taiwan dollars, of which 1 US$ = 30 NT$.

**Table 2:** Frequency distribution of traditional Chinese medicine (TCM) visits by major disease categories (according to 9th ICD codes) in subjects with insomnia ever been prescribed hypnotic drugs from 2002 to 2008 in Taiwan.

Diagnosis	ICD-9-CM codes	Treatment days (people)	
		Chinese herbal remedies	Acupuncture and manipulative therapies
Infectious and parasitic diseases	001–139	46,426 (2,027)	47 (14)
Neoplasms	140–239	63,080 (904)	777 (32)
Endocrine, nutritional, and metabolic diseases and immunity disorders	240–279	213,461 (4,425)	890 (90)
Psychotic diseases	290–319	164,603 (4,417)	1,781 (135)
Somnambulism	307.4	57,639 (2,073)	268 (34)
Others		106,964 (2,542)	1,513 (105)
Disease of nervous system and sense organs	320–389	332,372 (10,391)	6,057 (1,200)
Disease of circulation system	390–459	257,825 (6,088)	4,384 (288)
Disease of respiratory system	460–519	1,319,859 (26,741)	2,334 (253)
Disease of digestive system	520–579	1,349,092 (25,113)	3,501 (275)
Disease of genitourinary system	580–629	824,093 (15,653)	4,102 (189)
Disease of the skin and subcutaneous tissues	680–709	238,578 (8,315)	857 (72)
Disease of musculoskeletal system and connective tissue	710–739	647,207 (17,991)	135,465 (23,473)
Symptom, signs, and ill-defined conditions	780–799	3,036,297 (40,342)	11,642 (1,348)
Sleep disturbance	780.5	1,450,671 (35,386)	6,733 (590)
Others		1,585,626 (29,955)	4,909 (845)
Injury and poisoning	800–999	46,331 (3,324)	138,053 (26,466)
Supplementary classification	V01–V82, E800–E999	375 (30) 0 (0)	2 (2) 4 (1)
Others*		67,793 (2,570)	1,053 (353)

Total		8,607,392 (45,847)	310,943 (34,454)

*Include ranges of 280–289, 630–677, 740–759, and 760–779 ICD-9-CM code and missing data.

**Table 3:** The top five coprescribed Chinese formulas and sedative and hypnotic drugs for treating insomnia (ICD9: 307.4 or 780.5) between 2002 and 2008.

Chinese Medicine-sedative and hypnotic drugs	Total days of coprescribing	Total people of coprescribing	Average days of coprescribing Chinese and Western medicine (days/people)
Total			
Formulae	1,507,601	18,837	
* Jia-Wei-Xiao-Yao-San* (Augmented Rambling Powder)	70,708	4,029	17.5
* Suan-Zao-Ren-Tang* (Zizyphus Combination)	58,115	3,646	15.9
* Tian-Wang-Bu-Xin-Dan* (Ginseng and Zizyphus Combination)	46,040	2,744	16.8
* Chai-Hu-Jia-Long-Gu-Mu-Li-Tang* (Bupleurum and Mu Li Combination)	39,494	2,409	16.4
* Gan-Mai-Da-Zao-Tang* (Licorice and Jujube Combination)	38,158	2,097	18.2
Sedative and hypnotic drugs	2,041,718	19,994	
Zolpidem^1^	462,395	6,415	30.6
Alprazolam^1^	313,833	4,990	27.0
Lorazepam^1^	276,865	5,053	24.4
Estazolam^1^	180,646	2,400	31.3
Fludiazepam^1^	171,842	2,229	34.0

^1^Short-acting BZD (elimination half time ≤ 24 hours).

**Table 4:** Number (no.) of new cases, population-at-risk, and incidence rates and hazard ratios (HR); 95% confidence intervals (CI) for hip fracture estimated from multivariate Cox regression model on a random sample of the National Health Insurance Research Database among sample subjects and followed from 2002 to 2008.

Presence of hip fracture during the follow-up period	Hypnotic users without using Chinese medicine, no. cases/population	Hypnotic users coprescribed Chinese medicine, no. cases/population	Hypnotic users coprescribed Chinese medicine/hypnotic users without using Chinese medicine	
			HR	(95% CI)
*Numbers of hip fractures among hypnotic users *	152/5,848	294/19,994	0.57	(0.47–0.69)
≥30 days Incidence rate per 1,000 person-years	123/5,045 6.5	260/17,149 3.4	0.62	(0.50–0.77)
<30 days Incidence rate per 1,000 person-years	29/803 11.1	34/2,845 2.9	0.33	(0.20–0.54)
